# A systematic review of the predictors of disease progression in patients with autosomal dominant polycystic kidney disease

**DOI:** 10.1186/s12882-015-0114-5

**Published:** 2015-08-15

**Authors:** Claire Woon, Ashleigh Bielinski-Bradbury, Karl O’Reilly, Paul Robinson

**Affiliations:** Double Helix Consulting, Complete House, Macclesfield, Cheshire UK; Otsuka Pharmaceutical Europe Ltd, Wexham, UK

**Keywords:** Autosomal dominant polycystic kidney disease, Disease progression, Systematic review, End stage renal disease, Prognostic indicators

## Abstract

**Background:**

Autosomal dominant polycystic kidney disease (ADPKD) is a genetic disorder characterised by progressive renal cyst formation leading to renal failure in the majority of patients. The likelihood and rate of ADPKD progression is difficult to predict and there is a clear need to identify prognostic indicators that could be used to anticipate ADPKD progression, to aid the management of patients in clinical practice.

**Methods:**

A systematic literature review was conducted to identify publications detailing the natural history of ADPKD, including diagnosis, prognosis and progression. Publications were identified and filtered, and data were extracted, based on a predefined research protocol.

**Results:**

The review identified 2799 journal articles and 444 conference abstracts; 254 articles, including observational studies, clinical trials and reviews, proceeded to data extraction. Disease progression was associated with a variety of prognostic indicators, most commonly age and total kidney volume (TKV). In the identified clinical trials, the absence of a consistent measure of disease progression led to variation in the primary endpoints used. Consequently, there was difficulty in consistently and effectively demonstrating and comparing the efficacy of investigational treatments across studies. More consistency was found in the observational studies, where disease progression was most frequently measured by TKV and glomerular filtration rate.

**Conclusions:**

This systematic review identified age and TKV as the most commonly cited prognostic indicators in the published ADPKD literature. It is envisaged that this review may inform future research, trial design and predictive models of ADPKD natural history, helping to optimise patient care.

**Electronic supplementary material:**

The online version of this article (doi:10.1186/s12882-015-0114-5) contains supplementary material, which is available to authorized users.

## Background

Polycystic kidney disease (PKD) is the most common genetic disorder leading to end stage renal disease (ESRD). Autosomal dominant polycystic kidney disease (ADPKD) is caused by germline mutations in *PKD1* (85 % of cases) and *PKD2* (15 % of cases), and is typically diagnosed later in life than autosomal recessive polycystic kidney disease [1–4].

ADPKD is characterised by the progressive development of multiple bilateral renal cysts and has a prevalence of less than five patients per 10,000 of the population in the EU [5–7]. ADPKD is a systemic disease, with extra-renal manifestations including liver cysts, intracranial aneurysms and hypertension [3, 8]. Approximately 70 % of patients with ADPKD will progress to ESRD at a median age of 56 years [9, 10]. Patients with ADPKD may experience chronic pain, which may be debilitating, in addition to increased morbidity due to enlarged kidneys, which can lead to poor health-related quality of life (HRQoL) and reduced social interaction [3, 11].

ADPKD is associated with a high degree of clinical variability between patients, both within and between families, especially in terms of the likelihood and rate of progression towards ESRD. In the early stages of ADPKD, before renal function is significantly compromised, progression can be difficult to detect as patients are often asymptomatic. Currently, there is a lack of consensus in the published literature regarding the optimal factors for prediction of renal outcomes and the ideal variables that should be measured in order to monitor disease progression, especially in the early stages of the disease. This has led to difficulties in identifying patients at high risk of ESRD and in defining appropriate primary endpoints to consistently measure and compare the efficacy of therapies in development.

There is a requirement for greater understanding of the predictors of disease progression in ADPKD in order to optimise clinical trial design, treatment and patient care. The objective of this systematic review was to identify publications that detail the natural history of ADPKD, considering the indicators of early- and late-stage progression. It was envisaged that this review would aid clinical practice by informing future research and the development of a predictive model to estimate the likely rate of progression and ultimate long-term outcomes in patients with ADPKD.

## Methods

### Systematic literature search

A systematic review of the literature was conducted to identify publications that detail the natural history of ADPKD, including diagnosis, prognosis and progression. In the absence of a definitive measure of disease progression, all measures linked to the progression of ADPKD were considered, although it was anticipated that those linked to cyst development/renal enlargement and renal function would be the most relevant.

The systematic review was conducted and reported according to the principles in the Preferred Reporting Items for Systematic Reviews and Meta-Analyses (PRISMA) statement [12] (Additional file 1). A search was conducted in the following electronic databases:

- MEDLINE and MEDLINE In-Process
- Embase
- Cochrane Central Register of Controlled Trials (CENTRAL)
- The Cochrane Database of Systematic Reviews (CDSR)
- NHS Economic Evaluation Database (NHS EED)
- Health Economic Evaluations Database (HEED)
- BIOSIS.

Electronic databases were searched on 7th April 2014 using a structured search string, including terms for ADPKD and study type (Additional file 2). The search was restricted to publications from the previous 10 years in all databases except BIOSIS, which was limited to the past 3 years. Publications in the field of ADPKD have become significantly more numerous in recent years; therefore, it was considered that a sufficient number of relevant articles would be identified and that any earlier studies containing key information would be identified through reference explosion. In addition, diagnostic and prognostic techniques have progressed with the advancement of technology, with earlier publications often relying on subjective measures of progression, including kidney palpation.

‘Grey literature’ searches of relevant congresses, limited to the past 3 years, were also conducted (World Congress of Nephrology, American Society of Nephrology, American Academy of Nephrology, British Renal Society, European Renal Association and International Society for Pharmacoeconomics and Outcomes Research). Reference lists of systematic reviews and meta-analyses identified by the search were reviewed to identify additional relevant articles that were not identified in the systematic search due to study type restrictions.

Studies meeting the following criteria were considered for inclusion in the review:

- Study types: clinical trials, observational studies, systematic reviews, meta-analyses and non-systematic reviews
- Population: human, adults (≥18 years of age) with PKD, or ADPKD specifically
- Outcomes: diagnosis or prognosis
- Language: English.

### Study selection

Identified articles were screened by one reviewer, according to the criteria specified above. Articles were filtered using a positive exclusion method, whereby articles with insufficient information to warrant their exclusion remained in the review [13].

Initially, titles and abstracts of identified articles were reviewed (title/abstract screening) according to a checklist; full-text papers of the publications remaining after title/abstract screening were reviewed according to the same checklist (full text screening). Papers assessing HRQoL were excluded at full text screening if they did not report data on the progression of ADPKD.

Publications that included assessment of the link between ADPKD progression and genetic markers were only included in the review if they assessed the link with genotype (*PKD1*/*PKD2*). Publications assessing genetic markers at a molecular level, i.e. studies assessing specific single nucleotide polymorphisms, were excluded because this level of detail is not readily available to nephrologists in clinical evaluations. An independent reviewer checked a random selection of articles (10 %) at both title/abstract and full text screening to ensure consistency and accuracy.

### Data extraction

Data were extracted from studies that met the inclusion criteria. If more than one article was found to present data from the same study population, results were collated as appropriate.

Extracted data consisted of:

- Study characteristics, such as study design, duration and location
- Patient characteristics (see ‘Definitions’)
- Clinical characteristics and outcomes linked to disease progression (see ‘Definitions’)
- Information regarding ESRD in the patient populations, such as age at ESRD onset and length of time on dialysis.

### Definitions

For the purposes of this review, ‘patient characteristics’ were demographic data and ‘clinical characteristics’ were defined as baseline clinical parameters, baseline measures of renal size and function, and characteristics that were not influenced by changing renal parameters, e.g. genotype (Table 1). ‘Outcomes’ were defined as any measures or markers of disease progression, such as parameters reported over a time period, or measurements taken at both baseline and end of the study. The reporting of symptoms of ADPKD, such as pain, may have been considered as patient characteristics if only reported at baseline, but may also have been considered as outcomes if reported over time.

**Table 1:** Patient characteristics and outcomes in ADPKD

Patient characteristics	Clinical characteristics	Outcomes
Demographic factors: age, gender, BMI, weight, height, ethnicity	Baseline renal function parameters: GFR, serum creatinine, proteinuria, albuminuria, creatinine clearance, albumin/creatinine ratio	Change in renal function parameters: GFR, serum creatinine, proteinuria, albuminuria, creatinine clearance, albumin/creatinine ratio
	Baseline renal size parameters: TKV, TCV, TKL, left renal volume, right renal volume, height-adjusted TKV	Change in renal size parameters: TKV, TCV, TKL, left renal volume, right renal volume, height-adjusted TKV
	Clinical measures: presence of hypertension, renal blood flow, haematuria, UTI	Change in clinical measures: renal blood flow, hypertension
	Genotype	

*BMI* body mass index, *GFR* glomerular filtration rate, *TCV* total cyst volume, *TKL* total kidney length, *TKV* total kidney volume, *UTI* urinary tract infection

Glomerular filtration rate (GFR) was a recognised measure of renal function, and was either estimated based on serum creatinine concentration using equations such as the Modification of Diet in Renal Disease (MDRD) equation or the Chronic Kidney Disease Epidemiology Collaboration (CKD-EPI) equation (estimated GFR [eGFR]), or measured by creatinine clearance (measured GFR [mGFR]). For simplicity, we used the term ‘GFR’ to refer to studies that used either eGFR or mGFR or both, or where methods of determining GFR were not defined. Change in GFR was used as a measure of renal function and change in total kidney volume (TKV) was used for renal volume in this review.

## Results

**Fig. 1:**
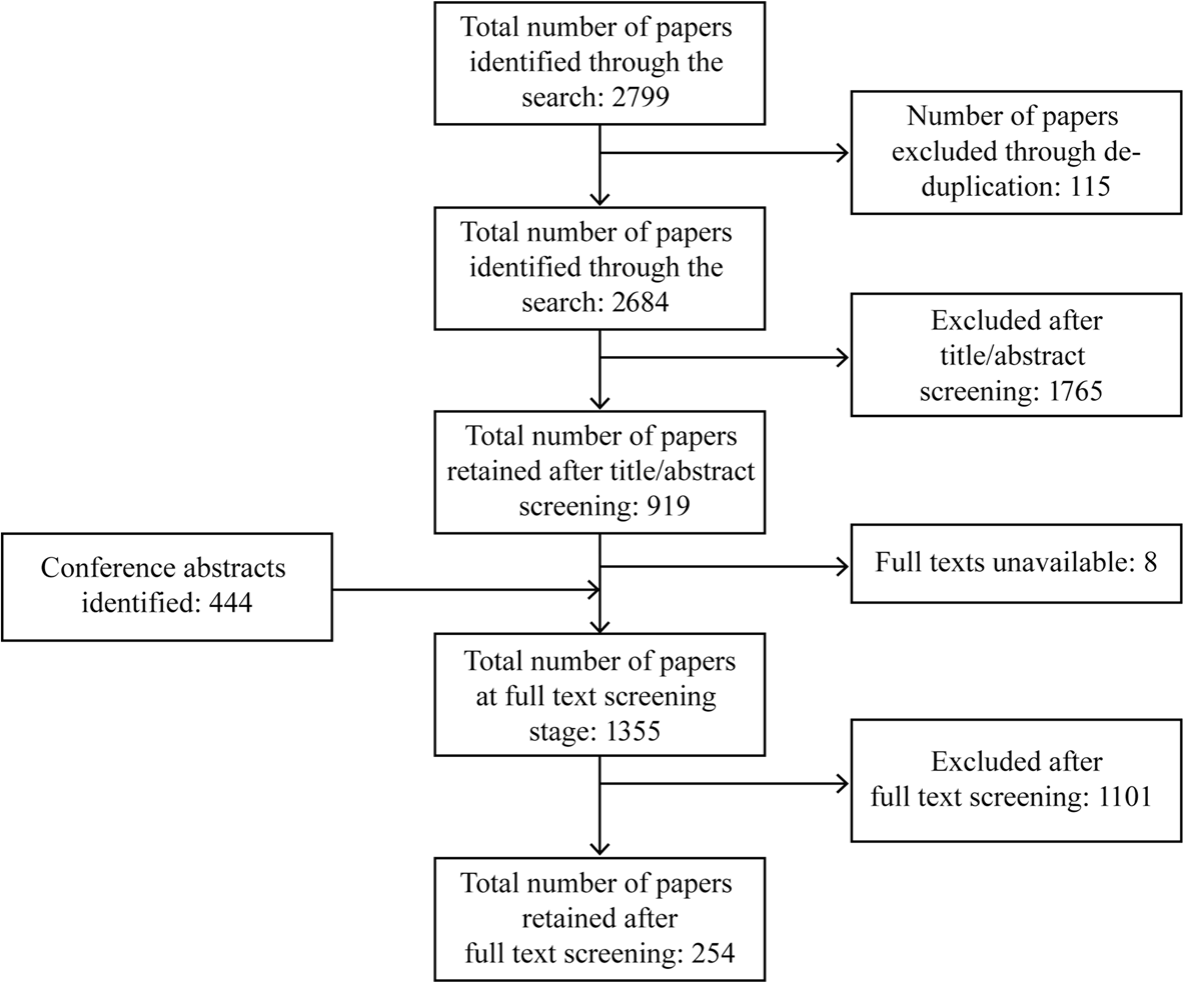
PRISMA diagram of the systematic review. PRISMA, Preferred Reporting Items for Systematic Reviews and Meta-Analyses

Figure 1 shows the selection process for articles included in the systematic review. A total of 2799 articles were identified in the original literature search, with a further 444 relevant conference abstracts identified. A total of 254 articles proceeded to the data extraction stage. Of the 254 papers that progressed to data extraction, 160 were observational studies, 33 were clinical trials and 61 were reviews.

### Observational studies

The majority of the identified studies were observational studies (63.0 %), including case studies, registry reviews and patient surveys with durations ranging from 6 months to more than 40 years. The studies employed a range of different objectives, with many aiming to increase understanding of ADPKD. Most studies reported baseline patient characteristics, including average age and gender (65 and 58.8 % of studies, respectively), while symptoms of ADPKD frequently reported at baseline included hypertension and proteinuria (21.3 and 10.6 % studies, respectively). The most commonly reported measure of renal function, described in 66 (41.3 %) observational studies, was GFR. The most frequent measure of renal size was TKV (34 studies, 21.3 %).

Of the 38 (23.8 %) publications that reported outcomes of ADPKD measured over time, disease progression was measured in 32 studies (84.2 %) by GFR, estimated most commonly by the MDRD equation (41.6 %). Change in TKV was reported as a measure of disease progression in 12 studies (31.6 %), with 11 publications using magnetic resonance imaging (MRI) and one publication using computed tomography. Renal enlargement over time as a surrogate marker of disease progression was also measured by total cyst volume (TCV) (five studies) [14–18], volume of each kidney (four studies) [19–22], and total renal volume (TRV) (three studies) [19, 22, 23].

In total, 19 studies reported patient and clinical characteristics of patients with ESRD. Of these studies, 18 reported age at ESRD and 11 reported the number of ADPKD patients reaching ESRD. Baseline characteristics of patients in each of the study groups were not reported in eight studies [24–31], but others reported a number of parameters including age (seven studies) [32–37], serum creatinine (four studies) [37–40], GFR (three studies) [33, 35, 38], TKV (three studies) [33, 36, 40], hypertension (one study) [41] and PKD mutation (one study) [42].

Publications were identified that measured GFR decline or increase in TKV (disease progression) and assessed the associated patient and clinical characteristics, defined in Table 1. If the association with patient and clinical characteristics was significant, these were then defined as prognostic indicators in the prediction of disease progression (Table 2).

**Table 2:** Summary of observational studies that identified prognostic indicators for ADPKD

Publication(s)	Objectives	Study details	Methodology for the assessment of disease progression and identification of prognostic indicators	Results and conclusions
Schrier, 2003 [40]	To assess the effect of increased research, identification of prognostic factors, and a higher number of anti-hypertensive medications on ADPKD progression	An observational study was conducted over two time periods, 255 patients from 1985 to 1992 (38 % male, age 37.5 ± 10 years, mean renal volume 701 cm^3^) and 258 patients from 1992 to 2001 (31 % male, age 37.5 ± 10 years, mean renal volume 704.5 cm^3^)	Regression analyses were performed for all patients to identify factors correlated with progression to ESRD, defined as dialysis or renal transplant	Age (*P* < 0.0001), renal volume (*P* < 0.0001), MAP (*P* = 0.0026) and UPE (*P* = 0.0051), but not gender, correlated with progression to ESRD
CRISP: Grantham, 2006; Torres, 2007, 2010, 2011; Harris, 2006; Irazabal, 2012; Marier, 2013 [15–17, 46, 48, 49, 55]	To identify markers of ADPKD disease progression	A prospective, long-term observational study of 241 ADPKD patients with normal renal function who were considered at high risk of renal insufficiency	Correlations between BL characteristics (including age, gender, BMI, hypertension, MAP, TKV, TCV, RBF, RVR, GFR, serum uric acid, Cho, 24-hour urine volume, UNaE, UAE, and estimated protein intake) and ∆TKV or ∆eGFR were assessed over 3, 6 or 8 years	Factors that predict ∆TKV:
				• BL TKV (*P* < 0.001)
				• BL RBF, UNaE, HDL-c, and age at BL (*P* ≤ 0.05)
				• Male gender (*P* = 0.0163)
				• UAE (when BL TKV was excluded, *P* ≤ 0.005)
				• There was no significant difference in ∆TKV or ∆TCV between patients with *PKD1* and *PKD2* mutations (*P* = 0.24 and 0.79, respectively)
				Factors that predict ∆GFR:
				• TKV (*P* < 0.02, 6 years’ follow-up). TKV for patients with BL TKV > 1500 mL (*P* < 0.001), but not for BL TKV < 750 mL (*P* = 0.063) or 750–1500 mL (*P* = 0.57, after 3 years of follow up)
				• Age (when BL GFR was excluded, *P* ≤ 0.02)
				• SCr, BUN (both *P* ≤ 0.001)
				• BL GFR, RBF and UNaE (*P* ≤ 0.02)
				• UAE, BSA and 24-h urine osmolarity (when BL GFR was excluded, *P* ≤ 0.02)
Kistler, 2009 [92]	To assess the reliability of TKV MRI imaging over 6 months with the aim of using such timescales in studies of potential treatments	A prospective study of 100 young patients with ADPKD (63 % male, mean age 31.2 ± 6.4 years, 97 % had family history of ADPKD) with preserved GFR	Correlations between % change in TKV and clinical and demographical parameters (not specified) were calculated to identify prognostic indicators	Higher ∆TKV were observed in males (*P* = 0.339) and in patients with albuminuria (*P* = 0.005)
Boertien, 2010, 2012 [93, 94]	To study the correlation between endogenous vasopressin concentration using plasma copeptin as a marker, and renal function	A review of data from a trial investigating the effect of an ACEI on progression of ADPKD (no treatment effect reported). Data were reviewed for 79 patients (43 % male, age 36.8 ± 10.1 years, GFR 96.8 ± 18.2 mL/min/1.73 m^2^) over a median of 11.2 years	∆eGFR was used as a measure of renal function	Higher baseline copeptin was associated with a faster ∆eGFR (*P* < 0.01). This association was independent of age, gender and BL eGFR
Azurmendi, 2011 [23]	To investigate albuminuria, measured by urinary Alb/Cr, as a predictor of disease progression	32 patients with ADPKD (mean age 26 ± 1 years) were observed over 30 ± 1 months	Yearly change in TKV, urinary MCP1 and eGFR were used as measures of renal function	In patients with high urinary Alb/Cr, yearly change in both TKV and urinary MCP1, but not eGFR, were increased compared with patients with normal urinary Alb/Cr (*P* < 0.05)
Griveas, 2012 [58]	To identify patients with ADPKD who progressed and those who did not	A retrospective review of 120 patients (39 % male, age 36.7 ± 12.7 years) was conducted with a median follow-up of 52 months	Correlations were made between annual change in eGFR and the following BL characteristics:	Higher BL eGFR was associated with a faster ∆eGFR (*P* = 0.04). Correlations between annual ∆eGFR and other BL characteristics were not significant
			• eGFR	
			• Hb	
			• Cho	
			• Parathormone	
			• SBP and DBP	
			• MAP	
Kurashige, 2012, 2013 [95, 96]	To investigate genotypic indicators of disease progression in Japanese patients with ADPKD	A mutation search was conducted in the coding and flanking regions of *PKD1/2* from 180 patients from 161 unrelated families	Multiple linear regression analyses were conducted to assess the correlations between eGFR decline and gene mutations, plasma arginine vasopressin, and urine osmolarity	• Patients with *PDK1* mutations had significantly faster ∆eGFR than patients with *PDK2* mutations (*P* < 0.01)
				• There was no association between ∆eGFR and mutation type or position
				• Lower urine osmolarity was found to correlate with ∆eGFR (significance not reported)
				• Plasma arginine vasopressin was significantly associated with ∆eGFR in patients with PDK1 mutations (*P* = 0.018)
Panizo, 2012 [60]	To analyse factors influencing ADPKD disease progression	A retrospective observational study was conducted in 101 patients with ADPKD (mean age 43 ± 17.3 years, 43.6 % male, median follow-up 69 months, mean kidney size 14.8 ± 2.9 cm, mean eGFR 74.5 ± 32.0 mL/min/1.73 m^2^)	The following data were collected as potential prognostic markers using eGFR reduction as an indicator of renal function decline:	• SBP, DBP, uric acid, total and LDL-c, Cr, microalbuminuria and kidney size were significantly associated with ∆eGFR (*P* ≤ 0.05)
			• Kidney size	• Younger age at diagnosis was also associated with rapid ∆eGFR (*P* = 0.010)
			• SBP and DBP	
			• Concomitant medications	
			• Hb	
			• Cr	
			• Uric acid	
			• Total Cho, HDL-c and LDL-c	
			• Triglycerides	
			• Calcium	
			• Phosphorus	
			• Parathyroid hormone	
			• Proteinuria (microalbuminuria)	
			• Haematuria	
CRISP: Warner, 2012 [56]	To assess the association between CPSA and decline in eGFR to determine whether this is a better indicator of ADPKD progression than TKV	Patients were randomly selected from the CRISP cohort: 10 rapid progressors, 10 slow progressors, and 4 atypical cases with large TKV and a small number of cysts at baseline^a^		• When the atypical cases were excluded from the analysis, BL lnCPSA and lnTKV correlated equally well with ∆eGFR over 6 years (*P* = 0.0003)
				• When atypical cases were included, baseline lnCPSA correlated better than lnTKV with ∆eGFR (*P* < 0.0001 and *P* = 0.0008, respectively)
Hwang, 2013 [97]	To investigate the association between asymptomatic pyuria and the development of UTIs and the deterioration of renal function	Retrospective case control study of 256 patients with ADPKD (52 % male, mean age 48.1 ± 12.8 years, mean eGFR 91.1 ± 29.2 mL/min/1.73 m^2^) in South Korea observed over 1 year	∆GFR was used as a measure of renal function	Patients with chronic asymptomatic pyuria, who were predominantly female (58.5 %) exhibited a significantly faster ∆GFR (*P* = 0.01) than patients without pyuria or with transient pyuria
Lacquaniti, 2013 [98]	To quantify the predictive potential of apelin (marker of vasopressin) and copeptin (antagonist of vasopressin signalling) in ADPKD disease progression	A prospective observational study of 52 patients with ADPKD (60 % male, mean age 43 ± 10 years, mean TKV 1057.3 ± 417.9 mL, mean mGFR 47.7 ± 35.6 mL/min/1.73 m^2^) and 50 matched healthy control patients (50 % male, mean age 40.3 ± 9.6 years, mean mGFR 116.6 ± 17 mL/min/1.73 m^2^) were followed for 24 months	Renal function was assessed by combination of ∆mGFR and ∆TKV (>5 % per year)	Concentrations of apelin < 68.5 pg/mL (*P* = 0.0002) and copeptin > 9.5 pmol/L (*P* = 0.02) were each associated with a faster decline in renal function
Ozkok, 2013 [59]	To investigate the importance of clinical characteristics and biochemical data on disease progression	323 patients with ADPKD (46 % male, mean age 53 ± 15 years) were followed for a mean of 100 ± 38 months	In Cox regression analysis, the following factors were assessed as potential predictors of ∆GFR:	Age, hypertension, hernia, proteinuria, and urinary stone were significantly associated with faster ∆GFR (*P* ≤ 0.04)
			• Age	
			• Gender	
			• BL SCr	
			• Smoking history	
			• History of hypertension	
			• Abdominal wall hernia	
			• Hepatic cyst	
			• Familial history of ADPKD	
			• Macroscopic haematuria	
			• Proteinuria	
			• Urinary stone	
			• Palpable kidney	
			• Use of ACEIs and/or ARBs	
Spithoven, 2013 [99]	To measure CCr(TS) in patients with ADPKD compared with healthy adults	A case control study of 125 patients with ADPKD (56 % male, mean age 40.4 ± 10.8 years, mean TKV 1470 mL, mean mGFR 77.7 ± 30.1 mL/min/1.73 m^2^) and 215 healthy controls (48 % male, mean age 53.1 ± 10.3 years, mean eGFR 97.7 ± 17.0 mL/min/1.73 m^2^)	CCr(TS) was used as a measure of renal function	• CCr(TS) was significantly higher for patients with ADPKD than for controls (*P* < 0.001), which may be due to cyst formation
				• In patients with ADPKD, CCr(TS) correlated with BMI (*P* = 0.003), BL mGFR (*P* = 0.03) and age (*P* = 0.07), but was not associated with TKV, female sex, filtration fraction, serum albumin, albuminuria (all P > 0.1)
Thong & Ong, 2013 [100]	To analyse the natural history of ADPKD progression	A retrospective study of 210 patients with ADPKD (48.6 % male, mean age 45.6 ± 16.2 years)	Regression analyses were performed to identify risk factors for ∆eGFR for 55 patients who had eGFR and kidney length measurements recorded over 5 years	∆eGFR was significantly associated with age at diagnosis and with mean kidney length (both *P* < 0.05). Gender, hypertension, haematuria, proteinuria, UTIs, and liver cysts were not significantly associated with renal function decline
Chen, 2014 [14]	To identify parameters that predict cyst growth and decline in renal function	A prospective, longitudinal observational study was performed in 541 Chinese patients with ADPKD and eGFR ≥30 mL/min/1.73 m^2^ (54 % male, mean age 39.7 ± 12.1 years, eGFR 100.4 ± 20.1 mL/min/1.73 m^3^) over a median follow-up of 14.3 ± 10.6 months	Analyses were performed for 279 patients with measurements for all variables of the correlation between yearly change in eGFR or yearly % growth in TKV and:	The following parameters correlated with yearly eGFR:
			• Age	• Age (*P* = 0.016)
			• Sex	• History of hypertension (*P* = 0.056)
			• Observation time	• Use of anti-hypertensive drugs (*P* = 0.102)
			• History of hypertension	• BL eGFR (*P* = 0.290)
			• Use of anti-hypertensive drugs	• Log_10_ Pr/Cr (*P* < 0.001)
			• BP	• Log_10_ BL TKV (*P* < 0.001)
			• Macrohaematuria	• BL thrombocyte count (*P* = 0.031)
			• BL eGFR	
			• Pr/Cr	
			• BL TKV	
			• BL thrombocyte count	The following parameters correlated significantly with yearly TKV:
				• Age (*P* < 0.001)
				• Male sex (*P* = 0.023)
				• Observation time (*P* = 0.072)
				• Use of anti-hypertensive drugs (*P* = 0.015)
				• DBP (*P* = 0.041)
				• BL eGFR (*P* = 0.173)
				• Log_10_ Pr/Cr (*P* = 0.050)
				• Log_10_ BL TKV (*P* = 0.092)
				• BL thrombocyte count (*P* = 0.042)
Higashihara, 2014 [101]	To assess the relationship between TKV and kidney function (measured by eGFR)	An observational study of 64 patients with ADPKD who completed ≥3 measurements and did not have any clinical conditions affecting kidney volume (33 % male, mean age 47.0 ± 14.1 years, mean TKV 1681.1 ± 1001.1 mL, mean eGFR 60.2 ± 27.38 mL/min/1.73 m^2^)	TKV, GFR, SCr, Cr clearance, UPE, and BP were measured over 5 years	• TKV, height-adjusted TKV, BSA-adjusted TKV and log-TKV significantly correlated with eGFR (all *P* < 0.0001)
				• BL TKV, age, and final eGFR were significantly associated with the yearly change in eGFR (*P* = 0.0349, *P* < 0.001, *P* = 0.0011, respectively), but the relationship between BL eGFR and the yearly change in eGFR was not significant (*P* = 0.4007)
				• Although there was no significant correlation between age and the TKV parameters investigated (P > 0.1), there was a significant relationship between age and both the yearly % change in TKV and change in log-TKV (*P* < 0.01)
				• There was a significant correlation between BL and final TKV and the yearly change in TKV (both *P* < 0.001)

*ACEI* angiotensin-converting enzyme inhibitor, *ADPKD* autosomal dominant polycystic kidney disease, *Alb/Cr* albumin/creatinine ratio, *ARB* angiotensin receptor blocker, *BL* baseline, *BMI* body mass index, *BP* blood pressure, *BSA* body surface area, *BUN* blood urea nitrogen, *CCr(TS)* tubular secretion of creatinine, *Cho* cholesterol, *CPSA* cyst parenchyma surface area, *Cr* creatinine, *CRISP* consortium for radiologic imaging studies in polycystic kidney disease, *DBP* diastolic blood pressure, *∆eGFR* rate of decline in estimated glomerular filtration rate, *∆TCV* rate of increase in total cyst volume, *∆TKV* rate of increase in total kidney volume, *eGFR* estimated glomerular filtration rate, *GFR* glomerular filtration rate, *Hb* haemoglobin, *HDL-c* high density lipoprotein cholesterol, *LDL-c* low density lipoprotein cholesterol, *MAP* mean arterial pressure, *MCP1* monocyte chemoattractant protein-1, *mGFR* measured glomerular filtration rate, *MRI* magnetic resonance imaging, *Pr/Cr* protein/creatinine ratio, *RBF* renal blood flow, *RVR* renal vascular resistance, *SBP* systolic blood pressure, *SCr* serum creatinine, *TCV* total cyst volume, *TKV* total kidney volume, *UAE* urine albumin excretion, *UNaE* urine sodium excretion, *UPE* urine protein excretion, *UTI* urinary tract infection

^a^Rapid and slow decline not defined

TKV and age at baseline were the two factors most commonly cited as significantly associated with a faster rate of ADPKD progression, each cited in ten studies (Table 3). A wide range of prognostic indicators (a total of 26) were reported, many of which were only reported by one study, indicating a lack of consensus in the literature on the parameters implicated in ADPKD progression.

**Table 3:** Publications that reported a correlation between prognostic indicators and change in renal function or volume

Prognostic indicator	Number of publications reporting prognostic indicator	Publications reporting a significant association between prognostic indicator and rate of decrease in renal function (GFR)	Publications reporting a significant association between prognostic indicator and rate of increase in renal volume
Age e.g. age at diagnosis (years)	10	Higashihara, 2014 [101], Chen, 2014 [14], Schrier 2003 [40], Thong, 2013 [100]^a^, Torres, 2007 [16]^a,b^, Panizo, 2012 [60]^a^, Ozkok, 2013 [59], Marier, 2013 [49], Spithoven, 2013 [99]	Torres, 2011 [15]
			Chen, 2014 [14]
			Higashihara, 2014 [101]
Baseline TKV e.g. log_10_ baseline TKV (cm^3^)	10	Higashihara, 2014 [101], Chen, 2014 [14]^d^, Grantham, 2006 [46]^e^, Schrier, 2003 [40]^f^, Warner, 2012 [56], Panizo, 2012 [60]^g^, Lacquaniti, 2013 [98]^h^, Marier, 2013 [49], Torres, 2007, 2011 [15, 16]^b^	Chen, 2014 [14]^d^
			Grantham, 2006 [46]^e^
			Higashihara, 2014 [101]^i^
			Torres, 2007, 2011 [15, 16]
Baseline GFR (either estimated or measured)	6	Higashihara, 2014 [101]^j^, Chen, 2014 [14], Griveas, 2012 [58], Spithoven, 2013 [99]^c^, Torres, 2007 [16], Lacquaniti, 2013 [98]^h^	Chen, 2014 [14]
Proteinuria/albuminuria e.g. baseline albuminuria (mg/L)	5	Schrier, 2003 [40], Torres, 2007 [16]^k^, Ozkok, 2013 [59], Panizo, 2012 [60]^l^	Kistler, 2009 [92]
			Torres, 2007 [16]^b^
Blood pressure e.g. mean arterial pressure (mmHg)	4	Schrier, 2003 [40]^m^, Panizo, 2012 [60]^n^, Chen, 2014 [14]^o,p^, Ozkok, 2013 [59]^o^	Chen, 2014 [14]^q^
Male gender	3	None	Harris, 2006 [17]
			Kistler, 2009 [92]
			Chen, 2014 [14]
Urine sodium concentration	3	Torres, 2007 [16]	Torres, 2007, 2011 [15, 16]
			Irazabal, 2012 [48]
Protein/creatinine ratio	2	Azurmendi, 2011 [23]^r^	Chen, 2014 [14]
		Chen, 2014 [14]	Azurmendi, 2011 [23]^s^
*PKD1* genotype	2	Kurashige, 2012, 2013 [95, 96]	None
Cholesterol e.g. serum HDL-c (mg/dL)	1	None	Torres, 2011 [15]^s^
Other^t,u^	14	Chen, 2014 [14], Grantham, 2006 [46], Hwang, 2013 [97], Panizo, 2012 [60], Ozkok, 2013 [59], Warner, 2012 [56], Kurashige, 2013 [96], Spithoven, 2013 [99]^c^, Lacquaniti, 2013 [98]^h^, Torres, 2007, 2010, 2011 [15, 16, 55]^b^, Boertien, 2010, 2012 [93, 94]	Chen, 2014 [14]

Publications of the same study have been grouped to prevent double-counting of patients

*ADH* antidiuretic hormone, *ΔeGFR* rate of decline in estimated glomerular filtration rate, *eGFR* estimated glomerular filtration rate, *GFR* glomerular filtration rate, *HDL* high density lipoprotein, *HDL-c* high density lipoprotein cholesterol, *MCP1* monocyte chemoattractant protein-1, *mGFR* measured glomerular filtration rate, *TKV* total kidney volume

^a^Younger age at diagnosis ^b^when baseline GFR excluded from regression analyses ^c^renal function measured by tubular secretion of creatinine ^d^log_10_ baseline TKV correlated with annual % growth in TKV ^e^baseline TKV >1500 mL ^f^renal volume; ^g^kidney size; ^h^renal function assessed by combination of decrease in mGFR and increase in TKV ^i^baseline and final TKV ^j^final eGFR (*P* = 0.001) but not baseline eGFR (*P* = 0.401) significantly correlated with annual ΔeGFR ^k^albuminuria; ^l^microalbuminuria; ^m^mean arterial pressure ^n^diastolic and systolic blood pressure ^o^history of hypertension ^p^intake of hypertensive drugs ^q^diastolic blood pressure and antihypertensive drug intake ^r^high urinary albumin/creatinine ratio ^s^serum HDL ^t^other prognostic indicators in studies assessing disease progression by measures of renal function include: baseline creatinine, body mass index, body surface area, chronic asymptomatic pyuria, cholesterol, cyst parenchyma surface area, copeptin levels, MCP-1, plasma ADH in *PKD1* patients, presence of hernia, renal blood flow, thrombocyte count, uric acid, urinary stone and urine osmolality ^u^other prognostic indicators in studies assessing disease progression by measures of renal volume include: apelin levels, copeptin levels, renal blood flow, and thrombocyte count

Seventeen publications from the Consortium for Radiologic Imaging Studies in Polycystic Kidney Disease (CRISP) study were identified by the systematic review [15–17, 43–56]. CRISP is a prospective, long-term observational study, including 241 ADPKD patients with normal renal function who were considered at high risk of renal insufficiency. The study was initiated to increase the knowledge and understanding of ADPKD prognosis by establishing reliable measures of disease progression [2]. Patients were diagnosed due to a positive family history (asymptomatic family screening) or based on signs and symptoms related to the disease. The study includes patients with defined hypertension diagnosed before the age of 35 years; ADPKD diagnosed *in utero* or in the first year of life; 24-h urinary protein excretion greater than 300 mg/day; or an episode of gross haematuria in men before the age of 30 years (CRISP I population) [57]. A range of measurements have thus far been reported, and patients have been stratified by several different criteria across the CRISP publications, demonstrating the relevance of a number of prognostic factors, such as gender and age at diagnosis (Table 2). CRISP has identified the importance of TKV as a key prognostic indicator, particularly in the early stages of ADPKD where significant renal enlargement can occur prior to the loss of renal function [2, 46]. The CRISP study is ongoing, with 203 patients having been re-enrolled in CRISP II from July 2007 [15].

Patients were stratified by rate of ADPKD progression, and groups defined as fast or slow progressors (large or small annual decline in GFR, respectively), in four observational studies (Additional file 3) [56, 58–60]. Although this concept is important in the clinical setting, there is no definitive conclusion in the literature as to how the rate of progression should be measured or stratified. Across the four studies, a range of prognostic indicators were reported, including initial GFR, high blood pressure, age at diagnosis and TKV at a given time point.

Disease severity was correlated with HRQoL in a single study [11]. In general, for patients with a lower GFR, Short Form-36 health survey scores were lower, indicating reduced HRQoL [11].

A research group in Brest, France, conducted an observational study assessing 26 clinical, biological and genetic variables (not specified) for use in a prognostic model. The model was based on data from 1017 patients with ADPKD, and was tested in a sub-population of 255 patients who had either reached ESRD or were aged over 60 years [41]. The presence of hypertension along with at least one urologic complication, or the presence of a truncating *PKD1* mutation was reported to be associated with poorest renal outcome [41].

### Clinical trials

The 33 clinical trial publications were reviewed to ensure the same data were not extracted from multiple reports of the same study. Of these 33, 24 unique reports were identified. The trials assessed the efficacy of three main drug types to slow the progression of ADPKD:

- Mammalian target of rapamycin inhibitors, e.g. sirolimus, everolimus and tacrolimus [61–67].
- Somatostatin analogues, e.g. somatostatin, lanreotide and octreotide [68–73].
- The vasopressin v2 receptor antagonist, tolvaptan [74–80].

Further drugs were:

- Eicosapentaenoic acid, an anti-inflammatory omega-3 fatty acid [81].
- Pravastatin, a statin [82].
- Anti-hypertensives, e.g. angiotensin-converting enzyme inhibitors (ACEIs) (enalapril or ramipril) [83, 84] or the beta-blocker metoprolol [84].

In the clinical trials assessing treatments to halt or slow the development of ADPKD, efficacy was assessed using measures of disease progression. Change in TKV or renal function, measured by GFR or serum creatinine concentration, were the most commonly used primary endpoints in the identified trials.

Other primary endpoints considered in studies of disease-modifying interventions in patients with ADPKD included change in liver volume, change in TCV and change in urinary fatty acid-binding protein. The number of different primary endpoints used in the identified trials demonstrated inconsistencies in the measurement of efficacy due to the paucity of data on the progression of ADPKD.

In the 21 publications that reported data at multiple time points, the most frequently reported outcomes were TKV (14 reports, 66.7 %), eGFR (12 reports, 57.1 %), serum creatinine (7 reports, 33.3 %) and systolic blood pressure (9 reports, 42.9 %). Of these, 12 studies reported data at two time points and eight studies reported data at three or four time points. Data were recorded at baseline and after 6 months in six studies [62, 65, 68, 71–73], at baseline and after 12 months in one study [70], at baseline and after 24 months in three studies [61, 66, 82], and at baseline and after 36 months in two studies [83, 84].

Due to the short study duration and lack of suitable sub-analyses or patient level data, disease progression data from these trials were not appropriate for the assessment of the prognostic potential of the variables measured at baseline.

Hypertension was targeted as a manifestation of ADPKD in the Halt Progression of Polycystic Kidney Disease (HALT-PKD) study, a clinical trial studying the intensive blockade of the renin-angiotensin system with combination ACEIs and angiotensin receptor blockers (ARBs). The study aimed to test the hypothesis that rigorous blood pressure control was more effective in slowing progression of renal disease in early ADPKD than moderate blood pressure control, by comparing the combination treatment to ACEI monotherapy alone. The primary endpoint was annual change in TKV, which was lower with rigorous blood pressure control than with moderate blood pressure control (*P* = 0.006). The rate of change in GFR was similar for the two treatment groups [85, 86].

### Reviews

The 61 non-systematic and systematic reviews and meta-analyses identified were mainly focussed around new and potential treatment options, as well as reviews of the genetics, pathophysiology and manifestations of the disease. In general, the identified reviews acknowledged and discussed a range of different prognostic indicators, but did not provide conclusive agreement on these factors.

## Discussion

Our review demonstrated that, as a result of the extensive clinical variability associated with ADPKD, a range of biological, genetic and clinical characteristics have been reported in the published literature for both the prediction and measurement of ADPKD progression. Currently, therapeutic options for ADPKD focus on management of the symptoms and complications of the disease since there is no available treatment to slow ADPKD progression. Stratification of patients based on their predicted rate of progression could improve symptom management. The uncertainty surrounding the clinical progression of the disease has also contributed to a paucity of clinical trials investigating potential interventions.

Due to the variability in the rate of ADPKD progression and the length of time taken for patients to reach ESRD, progression to ESRD is a poor candidate endpoint for use in clinical trials. Instead, predictors/indicators of time to disease progression have been used to assess the efficacy of treatments; however, a variety of different endpoints have been employed. This has led to problems in demonstrating conclusive efficacy and in comparing treatments across trials. As a result, no consensus has been reached regarding the optimal endpoints to assess the progression of ADPKD and the efficacy of potential therapies. A range of observational studies have attempted to address this, but there is inconsistency in the parameters reported and in the assessment of disease progression.

Studies evaluating ADPKD have generally been conducted in relatively small study populations, because the slow rate of progression and the lengthy period during which GFR is within the normal range limits the size of the population available for analysis. The CRISP observational study is a rich data source that has assessed several prognostic indicators in a relatively large cohort of patients with ADPKD, selected in order to study the clinical and radiological features of perceived fast progressors over a long follow-up period (6 years) [15, 46]. To assess the relationship between baseline TKV and renal growth over time, the cohort was stratified; a greater baseline TKV at a younger age was associated with a more rapid increase in TKV [46].

CRISP also illustrated that, although GFR does not typically change until the fourth or fifth decade of life, renal enlargement progresses significantly in the early stages of the disease, identifying large TKV as a key indicator of early disease progression [2, 46]. This provides evidence that early treatment may be important to slow or halt the progression of ADPKD before cyst development causes irreversible damage [46]. Other prognostic indicators of relevance included hypertension, gender and age at diagnosis, some of which correlate with those identified by other studies [2]. It is anticipated that these prognostic indicators will be used in the future evaluation of treatments that aim to slow the progression of ADPKD and will lead to improvement and standardisation of trial design [2]. However, the CRISP study also has limitations in that, compared with the general ADPKD population, CRISP enrolled a younger cohort with well-preserved renal function. Further analyses are required to validate these findings in the wider ADPKD population with a broader range of renal function and volumes.

Since the searches were conducted, three abstracts from the PKD Outcomes Consortium (PKDOC) have been presented [87–89]. The PKDOC dataset is the largest ADPKD dataset currently available and includes data from CRISP in addition to other study populations from the Mayo Clinic, Emory University and the University of Colorado [89]. These abstracts validate the use of TKV as an important prognostic indicator, identifying baseline TKV and baseline eGFR as prognostic biomarkers for both eGFR decline and progression to ESRD.

Our systematic review has identified a wide range of prognostic indicators that have been proposed or assessed in observational studies and clinical trials, but there is currently no agreement within the literature. The absence of long term patient level data from the identified clinical trials prevented correlations between variables measured at baseline and the rate of disease progression. In observational studies, age and TKV at baseline were the two factors most commonly cited as significantly associated with a faster rate of ADPKD progression. However, further research is required to validate these factors as true indicators of disease progression. Reported prognostic indicators may be complications of ADPKD that are exacerbated by disease progression, rather than factors driving the underlying progression of the disease, or may exhibit a cause-effect association, in which they both exacerbate and are worsened by the progression of the disease.

The specific genotype of patients with ADPKD has also generated interest as a potential indicator of disease progression rate. Although it is generally agreed in the literature that patients with *PKD1* mutations have larger kidneys and worse renal function compared to patients with *PKD2* mutations, few papers report the use of GFR and/or TKV to measure disease progression in patients stratified by genotype. Therefore, despite the large number of potential prognostic indicators identified, there may be an interaction between some, possibly enabling the collapse of several prognostic indicators into fewer key factors that are most informative. The two most commonly reported prognostic indicators identified by this review were age and TKV at baseline, which may encapsulate other factors such as *PKD* gene mutation, renal blood flow and baseline GFR.

Due to inter-patient heterogeneity in renal size and genetic profile, current diagnostic practices based on renal ultrasound and family histories are suboptimal. Recently, urinary proteomic and microRNA biomarkers have been identified that could be used as a non-invasive method of diagnosing patients with ADPKD [90, 91]. Long-term studies are ongoing to determine whether such markers also serve as prognostic indicators in ADPKD [90]. However, initial results are promising as a correlation was observed between the urinary peptide profile and height-adjusted TKV in patients with ADPKD [91].

The identification of prognostic indicators is of great importance in the drive to improve the management of ADPKD, as a better understanding of patients’ clinical prognosis may lead to improved symptom management that can be tailored to individuals, depending on their predicted rate of progression. This knowledge will also aid the stratification and selection of patients for disease-modifying treatments, as well as providing a basis for improved clinical trial design with standard endpoints to evaluate and compare treatments. A more thorough understanding of the natural history of ADPKD may be beneficial in terms of optimising treatment. The construction of a disease progression model to predict the progression of ADPKD, incorporating the identified prognostic indicators, could be informative for the future management of the disease.

## Conclusions

There has previously been little clinical consensus regarding the prognostic indicators associated with disease progression in ADPKD, especially in the early stages of the disease. As a result, ADPKD progression has been assessed using a wide range of patient and clinical characteristics. A systematic review of the literature has identified age and TKV as the two factors most commonly cited as significantly associated with a faster rate of ADPKD progression, particularly in the early stages of the disease. By identifying these prognostic indicators it is hoped there is an opportunity to improve trial design and conduct further research, including a predictive model of ADPKD natural history, in order to provide clinicians with a clearer understanding of factors influencing disease progression, thereby helping to optimise patient care.

## Additional files

Additional file 1:
**PRISMA 2009 checklist.** A checklist of items to be reported in systematic reviews and meta-analyses (DOCX 18 kb) Additional file 2:
**General search string.** General search string used to interrogate databases and identify potential studies of interest. (DOCX 17 kb) Additional file 3:
**Definitions of fast and slow progression.** Definitions of fast and slow progression of ADPKD used in observational studies. (DOCX 18 kb)

## Abbreviations

ACEI: Angiotensin-converting enzyme inhibitor
ADPKD: Autosomal dominant polycystic kidney disease
ARB: Angiotensin receptor blocker
CDSR: Cochrane database of systematic reviews
CENTRAL: Cochrane central register of controlled trials
CKD-EPI: Chronic kidney disease epidemiology collaboration
CRISP: Consortium for radiologic imaging studies in polycystic kidney disease
eGFR: Estimated glomerular filtration rate
ESRD: End stage renal disease
GFR: Glomerular filtration rate
HALT-PKD: Halt progression of polycystic kidney disease
HEED: Health economic evaluations database
HRQoL: Health-related quality of life
MDRD: Modification of diet in renal disease
mGFR: Measured glomerular filtration rate
MRI: Magnetic resonance imaging
NHS EED: NHS economic evaluation database
PKD: Polycystic kidney disease
PKDOC: Polycystic kidney disease outcomes consortium
PRISMA: Preferred reporting items for systematic reviews and meta-analyses
TCV: Total cyst volume
TKV: Total kidney volume
TRV: Total renal volume

## References

[1] Nahm AM, Henriquez DE, Ritz E (2002). Renal cystic disease (ADPKD and ARPKD). Nephrol Dial Transplant.

[2] Chapman AB (2008). Approaches to testing new treatments in autosomal dominant polycystic kidney disease: insights from the CRISP and HALT-PKD studies. Clin J Am Soc Nephrol.

[3] Luciano RL, Dahl NK (2014). Extra-renal manifestations of autosomal dominant polycystic kidney disease (ADPKD): considerations for routine screening and management. Nephrol Dial Transplant.

[4] Chapal M, Debout A, Dufay A, Salomon R, Roussey G, Burtey S, Launay EA, Vigneau C, Blancho G, Loirat C, Hourmant M, Fakhouri F (2012). Kidney and liver transplantation in patients with autosomal recessive polycystic kidney disease: a multicentric study. Nephrol Dial Transplant.

[5] Heidland A, Bahner U, Deetjen A, Gotz R, Heidbreder E, Schafer R, Teschner M (2009). Mass-screening for early detection of renal disease: benefits and limitations of self-testing for proteinuria. J Nephrol.

[6] Neumann HP, Jilg C, Bacher J, Nabulsi Z, Malinoc A, Hummel B, Hoffmann MM, Ortiz-Bruechle N, Glasker S, Pisarski P, Neeff H, Kramer-Guth A, Cybulla M, Hornberger M, Wilpert J, Funk L, Baumert J, Paatz D, Baumann D, Lahl M, Felten H, Hausberg M, Zerres K, Eng C (2013). Epidemiology of autosomal-dominant polycystic kidney disease: an in-depth clinical study for south-western Germany. Nephrol Dial Transplant.

[7] Patch C, Charlton J, Roderick PJ, Gulliford MC (2011). Use of antihypertensive medications and mortality of patients with autosomal dominant polycystic kidney disease: a population-based study. Am J Kidney Dis.

[8] Torres VE, Rossetti S, Harris PC (2007). Update on autosomal dominant polycystic kidney disease. Minerva Med.

[9] Shaw C, Simms RJ, Pitcher D, Sandford R (2014). Epidemiology of patients in England and Wales with autosomal dominant polycystic kidney disease and end-stage renal failure. Nephrol Dial Transplant.

[10] Spithoven EM, Kramer A, Meijer E, Orskov B, Wanner C, Caskey F, Collart F, Finne P, Fogarty DG, Groothoff JW, Hoitsma A, Nogier MB, Postorino M, Ravani P, Zurriaga O, Jager KJ, Gansevoort RT (2014). Analysis of data from the ERA-EDTA Registry indicates that conventional treatments for chronic kidney disease do not reduce the need for renal replacement therapy in autosomal dominant polycystic kidney disease. Kidney Int.

[11] Miskulin DC, Abebe KZ, Chapman AB, Perrone RD, Steinman TI, Torres VE, Bae KT, Braun W, Winklhofer FT, Hogan MC, Rahbari-Oskoui F, Moore CG, Flessner MF, Schrier RW (2014). Health-related quality of life in patients with autosomal dominant polycystic kidney disease and CKD stages 1–4: a cross-sectional study. Am J Kidney Dis.

[12] Moher D, Liberati A, Tetzlaff J, Altman DG (2009). Preferred reporting items for systematic reviews and meta-analyses: the PRISMA statement. Ann Intern Med.

[13] Higgins JPT, Green S. Cochrane handbook for systematic reviews of interventions Version 5.1.0. The Cochrane Collaboration. 2011. http://www.cochrane-handbook.org. Accessed 15 Jan 15 A.D.

[14] Chen D, Ma Y, Wang X, Yu S, Li L, Dai B, Mao Z, Sun L, Xu C, Rong S, Tang M, Zhao H, Liu H, Serra AL, Graf N, Liu S, Wuthrich RP, Mei C (2014). Clinical characteristics and disease predictors of a large Chinese cohort of patients with autosomal dominant polycystic kidney disease. PLoS One.

[15] Torres VE, Grantham JJ, Chapman AB, Mrug M, Bae KT, King BF, Wetzel LH, Martin D, Lockhart ME, Bennett WM, Moxey-Mims M, Abebe KZ, Lin Y, Bost JE (2011). Potentially modifiable factors affecting the progression of autosomal dominant polycystic kidney disease. Clin J Am Soc Nephrol.

[16] Torres VE, King BF, Chapman AB, Brummer ME, Bae KT, Glockner JF, Arya K, Risk D, Felmlee JP, Grantham JJ, Guay-Woodford LM, Bennett WM, Klahr S, Meyers CM, Zhang X, Thompson PA, Miller JP (2007). Magnetic resonance measurements of renal blood flow and disease progression in autosomal dominant polycystic kidney disease. Clin J Am Soc Nephrol.

[17] Harris PC, Bae KT, Rossetti S, Torres VE, Grantham JJ, Chapman AB, Guay-Woodford LM, King BF, Wetzel LH, Baumgarten DA, Kenney PJ, Consugar M, Klahr S, Bennett WM, Meyers CM, Zhang QJ, Thompson PA, Zhu F, Miller JP (2006). Cyst number but not the rate of cystic growth is associated with the mutated gene in autosomal dominant polycystic kidney disease. J Am Soc Nephrol.

[18] Irazabal-Mira MV, Torres VE, Hogan MC, Glockner J, King BF, Ofstie TG, Krasa HB, Ouyang J, Czerwiec FS (2010). Short-term effects of tolvaptan on renal function and volume in patients with autosomal dominant polycystic kidney disease (ADPKD). J Am Soc Nephrol.

[19] Ulusoy S, Ozkan G, Kosucu P, Kaynar K, Eyuboglu I (2012). A comparison of the effects of losartan and ramipril on blood pressure, renal volume and progression in polycystic kidney disease: A 5-Year follow-up. Hippokratia.

[20] Peces R, Cuesta-Lopez E, Peces C, Perez-Duenas V, Vega-Cabrera C, Selgas R (2011). Octreotide reduces hepatic, renal and breast cystic volume in autosomal-dominant polycystic kidney disease. Int U Nephrol.

[21] Rim H, Jung GS, Jung YS (2010). Transcatheter arterial embolization using ethanol in a dialysis patient for contracting enlarged polycystic kidneys. Korean J Radiol.

[22] Ulusoy S, Ozkan G, Orem C, Kaynar K, Kosucu P, Kiris A (2010). A comparison of the effects of ramipril and losartan on blood pressure control and left ventricle hypertrophy in patients with autosomal dominant polycystic kidney disease. Ren Fail.

[23] Azurmendi PJ, Fraga AR, Valdez MG, Arrizurieta E, Martin RS (2011). Early progression markers in autosomal dominant polycystic kidney disease. A longitudinal study in patients with normal GFR. J Am Soc Nephrol.

[24] Dicks E, Ravani P, Langman D, Davidson WS, Pei Y, Parfrey PS (2006). Incident renal events and risk factors in autosomal dominant polycystic kidney disease: a population and family-based cohort followed for 22 years. Clin J Am Soc Nephrol.

[25] Helal I, Gitomer BY, McFann K, Yan XD, Brosnahan GM, Schrier RW (2011). Serum uric acid and renal disease progression in autosomal dominant polycystic kidney disease. J Am Soc Nephrol.

[26] Helal I, Gitomer BY, McFann K, Tkachenko OO, Yan XD, Schrier RW (2012). Changing pattern of end-stage renal disease treatment in autosomal dominant polycystic kidney disease patient over time. J Am Soc Nephrol.

[27] Nishimura H, Ubara Y, Nakamura M, Nakanishi S, Sawa N, Hoshino J, Suwabe T, Takemoto F, Nakagawa M, Takaichi K, Tomikawa S (2009). Renal cell carcinoma in autosomal dominant polycystic kidney disease. Am J Kidney Dis.

[28] Orskov B, Christensen KB, Feldt-Rasmussen B, Strandgaard S (2012). Low birth weight is associated with earlier onset of end-stage renal disease in Danish patients with autosomal dominant polycystic kidney disease. Kidney Int.

[29] Orskov B, Sorensen V, Feldt-Rasmussen B, Strandgaard S (2012). Changes in causes of death and risk of cancer in Danish patients with autosomal dominant polycystic kidney disease and end-stage renal disease. Nephrol Dial Transplant.

[30] Spithoven EM, Kramer A, Wanner C, Jager KJ, Gansevoort RT (2013). Incidence of renal replacement therapy for ADPKD in Europe. J Am Soc Nephrol.

[31] Yoo DJ, Agodoa L, Yuan CM, Abbott KC, Nee R (2014). Risk of intracranial hemorrhage associated with autosomal dominant polycystic kidney disease in patients with end stage renal disease. BMC Nephrol.

[32] Barua M, Cil O, Paterson AD, Wang K, He N, Dicks E, Parfrey P, Pei Y (2009). Family history of renal disease severity predicts the mutated gene in ADPKD. J Am Soc Nephrol.

[33] Chang MY, Chen HM, Jenq CC, Lee SY, Chen YM, Tian YC, Chen YC, Hung CC, Fang JT, Yang CW, Wu-Chou YH (2013). Novel PKD1 and PKD2 mutations in Taiwanese patients with autosomal dominant polycystic kidney disease. J Hum Genet.

[34] Cornec-Le Gall E, Treguer L, Sawadogo T, Benarbia S, Le Meur Y (2013). Clinical factors predicting renal outcome in autosomal dominant polycystic kidney disease (ADPKD): results of the GENKYST registry. Nephrol Dial Transplant.

[35] Haynes R, Staplin N, Emberson J, Herrington G, Tomson C, Agodoa L, Tesar V, Levin A, Lewis D, Reith C, Baigent C, Landray MJ (2014). Evaluating the contribution of the cause of kidney disease to prognosis in CKD: results from the study of heart and renal protection (SHARP). Am J Kidney Dis.

[36] Helal I, McFann K, Reed B, Yan XD, Schrier RW, Fick-Brosnahan GM (2013). Serum uric acid, kidney volume and progression in autosomal-dominant polycystic kidney disease. Nephrol Dial Transplant.

[37] Nunes ACF, Milani V, Porsch DB, Rossato LB, Mattos CB, Roisenberg I, Barros EJG (2008). Frequency and clinical profile of patients with polycystic kidney disease in southern Brazil. Ren Fail.

[38] Fary Ka E, Seck SM, Niang A, Cisse MM, Diouf B (2010). Patterns of autosomal dominant polycystic kidney diseases in black Africans. Saudi J Kidney Dis Transpl.

[39] Romao EA, Moyses Neto M, Teixeira SR, Muglia VF, Vieira-Neto OM, Dantas M (2006). Renal and extrarenal manifestations of autosomal dominant polycystic kidney disease. Braz J Med Biol Res.

[40] Schrier RW, McFann KK, Johnson AM (2003). Epidemiological study of kidney survival in autosomal dominant polycystic kidney disease. Kidney Int.

[41] Cornec-Le Gall E, Hourmant M, Morin MP, Perrichot R, Charasse C, Siohan P, Audrezet MP, Ferec C, Le Meur Y (2013). A new algorithm to predict renal outcome in autosomal dominant polycystic kidney disease. J Am Soc Nephrol.

[42] Cornec-Le Gall E, Audrezet MP, Hourmant M, Morin MP, Grall-Jezequel A, Renaudineau E, Ferec C, Le Meur Y (2012). PKD1 mutation type, but not the mutation location, influences renal outcome in autosomal dominant polycystic kidney disease. J Am Soc Nephrol.

[43] Boertien WE, Meijer E, Li J, Bost JE, Struck J, Flessner MF, Gansevoort RT, Torres VE (2013). Relationship of copeptin, a surrogate marker for arginine vasopressin, with change in total kidney volume and GFR decline in autosomal dominant polycystic kidney disease: results from the CRISP cohort. Am J Kidney Dis.

[44] Chapman AB, Bost JE, Torres VE, Mrug M, Bae KT, Grantham JJ (2010). Cyst-dependent renal complications in autosomal dominant polycystic kidney disease. J Am Soc Nephrol.

[45] Chapman AB, Bost JE, Torres VE, Guay-Woodford L, Bae KT, Landsittel D, Li J, King BF, Martin D, Wetzel LH, Lockhart ME, Harris PC, Moxey-Mims M, Flessner M, Bennett WM, Grantham JJ (2012). Kidney volume and functional outcomes in autosomal dominant polycystic kidney disease. Clin J Am Soc Nephrol.

[46] Grantham JJ, Torres VE, Chapman AB, Guay-Woodford LM, Bae KT, King BF, Wetzel LH, Baumgarten DA, Kenney PJ, Harris PC, Klahr S, Bennett WM, Hirschman GN, Meyers CM, Zhang X, Zhu F, Miller JP (2006). Volume progression in polycystic kidney disease. N Engl J Med.

[47] Grantham JJ, Torres VE, Chapman AB, Bae KT, Tao C, Guay-Woodford LM, Harris PC, Mrug M, Bennett WM, Moxey-Mims M, Bost JE (2010). Urinary monocyte chemotactic protein-1 (MCP1) predicts progression in autosomal dominant polycystic kidney disease (ADPKD). J Am Soc Nephrol.

[48] Irazabal MV, Boertien WE, Landsittel D, Li J, Struck J, Flessner MF, Gansevoort RT, Torres VE (2012). Urine sodium excertion and plasma proANP as markers of disease progression in ADPKD. J Am Soc Nephrol.

[49] Marier JF, Gosselin NH, Chittenden JT, Czerwiec FS, Levy DI, Chapman AB, Gitomer BY, Torres VE, Dennis EH, Romero K, Miskulin D, Perrone RD (2013). Total kidney volume is a prognostic biomarker for worsening of kidney function in patients with autosomal dominant polycystic kidney disease. J Am Soc Nephrol.

[50] Marier JF, Mouksassi M, Jonsson F, Czerwiec FS, Levy DI, Chapman AB, Gitomer BY, Torres VE, Dennis EH, Romero K, Miskulin D, Perrone RD (2013). Total kidney volume is a prognostic biomerker for the progression to end-stage renal disease in patients with autosomal dominant polycystic kidney disease over 10 years. J Am Soc Nephrol.

[51] Mrug M, Mrug S, Guay-Woodford LM, Torres VE, Bae KT, Harris PC, Landsittel D, Flessner MF, Bennett WM, Grantham JJ, Chapman AB (2012). Prediction of renal function trajectories in early autosomal dominant polycystic kidney disease. J Am Soc Nephrol.

[52] Mrug M, Mrug S, Landsittel D, Torres VE, Bae KT, Harris PC, Guay-Woodford LM, Flessner MF, Bennett WM, Grantham JJ, Chapman AB (2013). Prediction of GFR endpoints in early autosomal dominant polycystic kidney disease. J Am Soc Nephrol.

[53] Parikh CR, Dahl NK, Chapman AB, Bost JE, Edelstein CL, Comer DM, Zeltner R, Tian X, Grantham JJ, Somlo S (2012). Evaluation of urine biomarkers of kidney injury in polycystic kidney disease. Kidney Int.

[54] Rule AD, Torres VE, Chapman AB, Grantham JJ, Guay-Woodford LM, Bae KT, Klahr S, Bennett WM, Meyers CM, Thompson PA, Miller JP (2006). Comparison of methods for determining renal function decline in early autosomal dominant polycystic kidney disease: the consortium of radiologic imaging studies of polycystic kidney disease cohort. J Am Soc Nephrol.

[55] Torres VE, Chapman AB, King BF, Martin DR, Grantham JJ, Mrug M, Bae KT, Bennett WM, Moxey-Mims MM, Jie L, Bost JE (2010). Renal blood flow (RBF) is an underestimated tool to monitor the progression of autosomal dominant polycystic kidney disease (ADPKD). J Am Soc Nephrol.

[56] Warner JD, Irazabal MV, Erickson BJ, King BF, Bae KT, Grantham JJ, Chapman AB, Mrug M, Landsittel D, Flessner MF, Bennett WM, Torres VE (2012). A new metric to predict autosomal dominant polycystic kidney disease (ADPKD) progression: cyst parenchyma surface area (CPSA). J Am Soc Nephrol.

[57] Chapman AB, Guay-Woodford LM, Grantham JJ, Torres VE, Bae KT, Baumgarten DA, Kenney PJ, King BF, Glockner JF, Wetzel LH, Brummer ME, O’Neill WC, Robbin ML, Bennett WM, Klahr S, Hirschman GH, Kimmel PL, Thompson PA, Miller JP (2003). Renal structure in early autosomal-dominant polycystic kidney disease (ADPKD): The Consortium for Radiologic Imaging Studies of Polycystic Kidney Disease (CRISP) cohort. Kidney Int.

[58] Griveas I, Bishop K, World M (2012). Adult polycystic kidney disease: who needs hospital follow-up?. Artif Organs.

[59] Ozkok A, Akpinar TS, Tufan F, Kanitez NA, Uysal M, Guzel M, Caliskan Y, Alisir S, Yazici H, Ecder T (2013). Clinical characteristics and predictors of progression of chronic kidney disease in autosomal dominant polycystic kidney disease: a single center experience. Clin Exp Nephrol.

[60] Panizo N, Goicoechea M, Garcia de Vinuesa S, Arroyo D, Yuste C, Rincon A, Verdalles U, Ruiz-Caro C, Quiroga B, Luno J (2012). Chronic kidney disease progression in patients with autosomal dominant polycystic kidney disease. Nefrologia.

[61] Mora FP, Codianni P, Liern M, Grammatico D, Vallejo G (2013). Use of rapamycin to reduce the pathologic kidney volume growth in autosomal polycystic kidney disease. Pediatr Nephrol.

[62] Perico N, Antiga L, Caroli A, Ruggenenti P, Fasolini G, Cafaro M, Ondei P, Rubis N, Diadei O, Gherardi G, Prandini S, Panozo A, Bravo RF, Carminati S, De Leon FR, Gaspari F, Cortinovis M, Motterlini N, Ene-Iordache B, Remuzzi A, Remuzzi G (2010). Sirolimus therapy to halt the progression of ADPKD. J Am Soc Nephrol.

[63] Qian Q, Du H, King BF, Kumar S, Cosio FG, Torres VE (2007). Sirolimus reduces polycystic liver volume in ADPKD patients after renal transplantation. J Am Soc Nephrol.

[64] Serra AL, Poster D, Kistler AD, Krauer F, Raina S, Young J, Rentsch KM, Spanaus KS, Senn O, Kristanto P, Scheffel H, Weishaupt D, Wuthrich RP (2010). Sirolimus and kidney growth in autosomal dominant polycystic kidney disease. N Engl J Med.

[65] Soliman AR, Ismail E, Zamil S, Lotfy A (2009). Sirolimus therapy for patients with adult polycystic kidney disease: a pilot study. Transplant Proc.

[66] Stallone G, Infante B, Grandaliano G, Bristogiannis C, Macarini L, Mezzopane D, Bruno F, Montemurno E, Schirinzi A, Sabbatini M, Pisani A, Tataranni T, Schena FP, Gesualdo L (2012). Rapamycin for treatment of type I autosomal dominant polycystic kidney disease (RAPYD-study): a randomized, controlled study. Nephrol Dial Transplant.

[67] Walz G, Budde K, Mannaa M, Nurnberger J, Wanner C, Sommerer C, Kunzendorf U, Banas B, Horl WH, Obermuller N, Arns W, Pavenstadt H, Gaedeke J, Buchert M, May C, Gschaidmeier H, Kramer S, Eckardt KU (2010). Everolimus in patients with autosomal dominant polycystic kidney disease. N Engl J Med.

[68] Caroli A, Antiga L, Cafaro M, Fasolini G, Remuzzi A, Remuzzi G, Ruggenenti P (2010). Reducing polycystic liver volume in ADPKD: effects of somatostatin analogue octreotide. Clin J Am Soc Nephrol.

[69] Caroli A, Perico N, Perna A, Antiga L, Brambilla P, Pisani A, Visciano B, Imbriaco M, Messa P, Cerutti R, Dugo M, Cancian L, Buongiorno E, De Pascalis A, Gaspari F, Carrara F, Rubis N, Prandini S, Remuzzi A, Remuzzi G, Ruggenenti P (2013). Effect of longacting somatostatin analogue on kidney and cyst growth in autosomal dominant polycystic kidney disease (ALADIN): a randomised, placebo-controlled, multicentre trial. Lancet.

[70] Chrispijn M, Nevens F, Gevers TJG, Vanslembrouck R, van Oijen MGH, Coudyzer W, Hoffmann AL, Dekker HM, de Man RA, van Keimpema L, Drenth JPH (2012). The long-term outcome of patients with polycystic liver disease treated with lanreotide. Aliment Pharmacol Ther.

[71] Gevers TJG, Hol JC, Monshouwer R, Dekker H, Wetzels JF, Drenth JPH (2013). Lanreotide halts polycystic liver and kidney growth in patients with autosomal dominant polycystic kidney disease. J Am Soc Nephrol.

[72] Ruggenenti P, Remuzzi A, Ondei P, Fasolini G, Antiga L, Ene-Iordache B, Remuzzi G, Epstein FH (2005). Safety and efficacy of long-acting somatostatin treatment in autosomal-dominant polycystic kidney disease. Kidney Int.

[73] van Keimpema L, Nevens F, Vanslembrouck R, van Oijen MG, Hoffmann AL, Dekker HM, De Man RA, Drenth JP (2009). Lanreotide reduces the volume of polycystic liver: a randomized, double-blind, placebo-controlled trial. Hepatology.

[74] Boertien WE, Meijer E, de Jong PE, Bakker SJL, Czerwiec FS, Struck J, Oberdhan D, Shoaf SE, Krasa HB, Gansevoort RT (2013). Short-term renal hemodynamic effects of tolvaptan in subjects with autosomal dominant polycystic kidney disease at various stages of chronic kidney disease. Kidney Int.

[75] Czerwiec FS, Chapman AB, Devuyst O, Gansevoort RT, Higashihara E, Krasa HB, Ouyang J, Perrone RD, Torres VE (2013). Clinical outcomes in ADPKD: results from the TEMPO 3:4 trial. J Am Soc Nephrol.

[76] Horie S, Higashihara E, Muto S, Nutahara K, Iino Y, Narita I, Ouyang J, Torres VE (2013). Effects of tolvaptan in ADPKD: subanalysis of Japanese patients from the TEMPO 3:4 trial. J Am Soc Nephrol.

[77] Perrone RD, Chapman AB, Czerwiec FS, Devuyst O, Gansevoort RT, Grantham JJ, Higashihara E, Krasa HB, Ouyang J, Torres VE (2013). Correlation of total kidney volume and eGFR in patients with ADPKD: results from the TEMPO 3:4 trial. J Am Soc Nephrol.

[78] Torres VE, Grantham JJ, Chapman AB, Watnick T, Kedzierski K, Ouyang JJ, Orlandi C, Czerwiec FS (2007). Phase 2 open-label study to determine safety, tolerability and efficacy of split-dose tolvaptan in ADPKD [abstract no:SA-PO077]. J Am Soc Nephrol.

[79] Torres VE, Chapman AB, Grantham JJ, Watnick TJ, Ouyang J, Krasa HB, Czerwiec FS (2010). TEMPO 2/4 update: changes in ADPKD total kidney volume and eGFR with 3 years of tolvaptan and after withdrawal. J Am Soc Nephrol.

[80] Torres VE, Chapman AB, Devuyst O, Gansevoort RT, Grantham JJ, Higashihara E, Perrone RD, Krasa HB, Ouyang J, Czerwiec FS, Trial I (2012). Tolvaptan in patients with autosomal dominant polycystic kidney disease. N Engl J Med.

[81] Higashihara E, Nutahara K, Horie S, Muto S, Hosoya T, Hanaoka K, Tuchiya K, Kamura K, Takaichi K, Ubara Y, Itomura M, Hamazaki T (2008). The effect of eicosapentaenoic acid on renal function and volume in patients with ADPKD. Nephrol Dial Transplant.

[82] Fassett RG, Coombes JS, Packham D, Fairley KF, Kincaid-Smith P (2010). Effect of pravastatin on kidney function and urinary protein excretion in autosomal dominant polycystic kidney disease. Scand J Urol Nephrol.

[83] van Dijk MA, Breuning MH, Duiser R, van Es LA, Westendorp RG (2003). No effect of enalapril on progression in autosomal dominant polycystic kidney disease. Nephrol Dial Transplant.

[84] Zeltner R, Poliak R, Stiasny B, Schmieder RE, Schulze BD (2008). Renal and cardiac effects of antihypertensive treatment with ramipril vs metoprolol in autosomal dominant polycystic kidney disease. Nephrol Dial Transplant.

[85] Schrier RW, Abebe KZ, Perrone RD, Torres VE, Braun WE, Steinman TI, Winklhofer FT, Brosnahan G, Czarnecki PG, Hogan MC, Miskulin DC, Rahbari-Oskoui FF, Grantham JJ, Harris PC, Flessner MF, Bae KT, Moore CG, Chapman AB (2014). Blood pressure in early autosomal dominant polycystic kidney disease. N Engl J Med.

[86] Torres VE, Abebe KZ, Chapman AB, Schrier RW, Braun WE, Steinman TI, Winklhofer FT, Brosnahan G, Czarnecki PG, Hogan MC, Miskulin DC, Rahbari-Oskoui FF, Grantham JJ, Harris PC, Flessner MF, Moore CG, Perrone RD (2014). Angiotensin blockade in late autosomal dominant polycystic kidney disease. N Engl J Med.

[87] Perrone R, Marier JF, Mouksassi M, Romero K, Dennis EH, Miskulin D, Czerwiec FS, Gitomer BY, Chapman A, Torres V (2014). Baseline total kidney volume is associated with worsening of kidney function independently of baseline glomerular filtration rate in patients with autosomal dominant polycystic kidney disease. Abstract 318 presented at the National Kidney Foundation Spring Clinical Meeting, Las Vegas, Nevada, USA, 22–26 Apr 2014.

[88] Perrone R, Marier JF, Mouksassi M, Czerwiec FS, Romero K, Dennis EH, Miskulin D, Chapman A, Gitomer BY, Torres V (2014). End-stage renal disease in patients with autosomal dominant polycystic kidney disease is dependent on baseline total kidney volume and baseline glomerular filtration rate. Abstract 319 presented at the National Kidney Foundation Spring Clinical Meeting, Las Vegas, Nevada, USA, 22–26 Apr 2014.

[89] Perrone R, Marier JF, Mouksassi M, Czerwiec FS, Romero K, Dennis EH, Miskulin D, Chapman A, Gitomer BY, Torres V (2014). Qualification of total kidney volume as a prognostic biomarker for use in clinical trials evaluating patients with autosomal dominant polycystic kidney disease. Abstract 428 presented at the National Kidney Foundation Spring Clinical Meeting, Las Vegas, Nevada, USA, 22–26 Apr 2014.

[90] Ben-Dov IZ, Tan YC, Morozov P, Wilson PD, Rennert H, Blumenfeld JD, Tuschl T (2014). Urine microRNA as potential biomarkers of autosomal dominant polycystic kidney disease progression: description of miRNA profiles at baseline. PLoS One.

[91] Kistler AD, Serra AL, Siwy J, Poster D, Krauer F, Torres VE, Mrug M, Grantham JJ, Bae KT, Bost JE, Mullen W, Wuthrich RP, Mischak H, Chapman AB (2013). Urinary proteomic biomarkers for diagnosis and risk stratification of autosomal dominant polycystic kidney disease: a multicentric study. PLoS One.

[92] Kistler AD, Poster D, Krauer F, Weishaupt D, Raina S, Senn O, Binet I, Spanaus K, Wuthrich RP, Serra AL (2009). Increases in kidney volume in autosomal dominant polycystic kidney disease can be detected within 6 months. Kidney Int.

[93] Boertien WE, Meijer E, Jie L, Bost JE, Struck J, Flessner MF, Gansevoort RT, Torres VE (2011). Copeptin, a surrogate marker for vasopressin, is associated with disease progression in the CRISP cohort of ADPKD patients. J Am Soc Nephrol.

[94] Boertien WE, Meijer E, Zittema D, van Dijk MA, Rabelink TJ, Breuning MH, Struck J, Bakker SJL, Peters DJM, de Jong PE, Gansevoort RT (2012). Copeptin, a surrogate marker for vasopressin, is associated with kidney function decline in subjects with autosomal dominant polycystic kidney disease. Nephrol Dial Transplant.

[95] Kurashige M, Hanaoke K, Kawaguchi Y, Hasegawa T, Imamura M, Maeda S, Hosoya T (2012). Genetic and phenotypic characteristics of subjects with autosomal dominant polycystic kidney disease in the Japanese. J Am Soc Nephrol.

[96] Kurashige M, Hanaoka K, Imamura M, Kawaguchi Y, Hasegawa E, Hosoya T, Maeda S, Yokoo T (2013). A comprehensive mutation search within the PKD1/2 for Japanese subjects with autosomal dominant polycystic kidney disease. J Am Soc Nephrol.

[97] Hwang JH, Park HC, Jeong JC, Ha Baek S, Han MY, Bang K, Cho JY, Yu SH, Yang J, Oh KH, Hwang YH, Ahn C (2013). Chronic asymptomatic pyuria precedes overt urinary tract infection and deterioration of renal function in autosomal dominant polycystic kidney disease. BMC Nephrol.

[98] Lacquaniti A, Chirico V, Lupica R, Buemi A, Loddo S, Caccamo C, Salis P, Bertani T, Buemi M (2013). Apelin and copeptin: two opposite biomarkers associated with kidney function decline and cyst growth in autosomal dominant polycystic kidney disease. Peptides.

[99] Spithoven EM, Meijer E, Boertien WE, Sinkeler SJ, Tent H, de Jong PE, Navis G, Gansevoort RT (2013). Tubular secretion of creatinine in autosomal dominant polycystic kidney disease: consequences for cross-sectional and longitudinal performance of kidney function estimating equations. Am J Kidney Dis.

[100] Thong KM, Ong ACM (2013). The natural history of autosomal dominant polycystic kidney disease: 30-year experience from a single centre. QJM.

[101] Higashihara E, Nutahara K, Okegawa T, Shishido T, Tanbo M, Kobayasi K, Nitadori T (2014). Kidney volume and function in autosomal dominant polycystic kidney disease. Clin Exp Nephrol.

