# Supplementary material for: A systematic review of the predictors of disease progression in patients with autosomal dominant polycystic kidney disease
Source: BMC Nephrol. 2015 Aug 15;16:140. doi: 10.1186/s12882-015-0114-5 (PMC4536696; doi:10.1186/s12882-015-0114-5)
Supplement: Additional file 1: — PRISMA 2009 checklist. A checklist of items to be reported in systematic reviews and meta-analyses (DOCX 18 kb) [file 12882_2015_114_MOESM1_ESM.docx]

**Additional file 1: General search string**

| 1 | “Randomised Controlled Trial”[Publication Type] OR “Randomised Controlled Trials”[MeSH] OR “randomised controlled trial” OR randomized controlled trial” |
| --- | --- |
| 2 | “Random Allocation”[MeSH] OR Random* NEAR (allocate* OR allot* OR assign* OR basis* OR divid* OR order*) |
| 3 | “Controlled Clinical Trial”[Publication Type] OR “Controlled Clinical Trials”[MeSH] OR “controlled clinical trial” |
| 4 | (clinical OR controlled OR comparative OR placebo OR prospective OR randomised OR randomised) NEAR (trial OR study) |
| 5 | “Clinical Trial”[Publication Type] OR “Clinical Trials”[MeSH] OR clinical trial* OR “Clinical Trial, Phase II”[Publication Type] OR “Clinical Trial, Phase III”[Publication Type] OR “Clinical Trial, Phase IV”[Publication Type] OR phase II clinical trial* OR phase III clinical trial* OR phase IV clinical trial* OR phase 2 clinical trial* OR phase 3 clinical trial* OR phase 4 clinical trial* |
| 6 | “Double-blind-method”[MeSH] OR “double-blind” OR “double blind” OR “Single-blind-method”[MeSH] OR “single-blind” OR “single blind” |
| 7 | (Singl* OR doubl*) NEAR (blind* OR mask*) |
| 8 | #1 OR #2 OR #3 OR #4 OR #5 OR #6 OR #7 |
| 9 | “Meta-Analysis”[Publication Type] OR “Meta-Analysis”[MeSH] OR meta-analys* OR meta analys* |
| 10 | Systematic NEAR (research OR review OR search OR overview) |
| 11 | Synthes* NEAR (literature OR research OR studies OR study OR data) |
| 12 | (Review OR research) SAME (systematic* OR methodologic* OR quantitative OR effective*) |
| 13 | “Systematic review” |
| 14 | Observational study* OR observational trial* OR “case control” OR “case controlled” OR case-control* OR cohort-studies[MeSH] OR cross-over-studies[MeSH] OR (naturalistic pre/0 (study or studies or trial or trials)) OR (("Real-world" or "real world") pre/0 (study or studies or trial or trials)) |
| 15 | #9 OR #10 OR #11 OR #12 OR #13 OR #14 |
| 16 | "Letter"[Publication Type] OR "Editorial"[Publication Type] OR "Comment"[Publication Type] OR "Review of Reported Cases"[Publication Type] OR "Review, Tutorial"[Publication Type] OR "Review, Multicase"[Publication Type] OR “Case-control-studies”[MeSH] |
| 17 | (#8 OR #15) NOT #16 |
| 18 | “Economic*” OR “Costs” OR “Costing” OR “Cost” OR “Costed” OR “Cost*” OR “Cost-Benefit*” OR “Cost-Effective*” OR “Cost-Utility” |
| 19 | “Patient Related Cost*” OR “Burden” OR “Cost Of Treat*” OR “Pharmacoeconomic*” OR “Illness Cost*” |
| 20 | (“Costs” And “Cost-Analysis”[MeSH]) OR (“Cost-Of-Illness”[MeSH] OR “Economics”[MeSH]) |
| 21 | “Cost-Benefit-Analysis”[MeSH] |
| 22 | “Economics-Hospital”[MeSH] OR “Economics-Nursing”[MeSH] OR “Economics-Pharmaceutical”[MeSH] |
| 23 | (“Direct” OR “Indirect” OR “Healthcare”) NEAR (“Cost” OR “Costs”) |
| 24 | “Cost-Consequence” OR “Cost Consequence” |
| 25 | #18 OR #19 OR #20 OR #21 OR #22 OR #23 OR #24 |
| 26 | “polycystic kidney disease”[MeSH] OR “Polycystic Kidney, Autosomal Dominant”[MESH] OR (polycystic AND kidney) |
| 27 | "autosomal dominant polycystic kidney disease" OR adpkd OR "cystic kidney" OR "cystic kidney disease" OR "kidney multicystic aplasia" OR "kidney multicystic disease" OR "kidney polycystosis" OR "polycystic kidney" OR "polycystic renal disease" OR "renal cystic disease" OR "renal polycystic disease" |
| 28 | #26 OR#27 |
| 29 | “animals”[MeSH] |
| 30 | “humans”[MeSH] |
| 31 | #29 NOT (#29 AND #30) |
| 32 | (#28 AND #17) NOT #31 |
| 33 | (#25 AND #28) NOT #31 |
