# Supplementary material for: A systematic review of the predictors of disease progression in patients with autosomal dominant polycystic kidney disease
Source: BMC Nephrol. 2015 Aug 15;16:140. doi: 10.1186/s12882-015-0114-5 (PMC4536696; doi:10.1186/s12882-015-0114-5)
Supplement: Additional file 2: — General search string. General search string used to interrogate databases and identify potential studies of interest. (DOCX 17 kb) [file 12882_2015_114_MOESM2_ESM.docx]

**Additional file 2:** **Definitions of fast and slow progression**

| **Author, year** | **Definition** | | **Units** |
| --- | --- | --- | --- |
|  | **Fast progressor** | **Slow progressor** |  |
| Ozkok, 2013 [59] | CKD-EPI eGFR >1 | CKD-EPI eGFR <1 | mL/min/year |
| Griveas, 2012 [58] | Patients with a statistically significant^a^ annual decline in eGFR^b^ | Patients without a statistically significant^a^ annual decline in eGFR^c^ | N/A |
| Panizo, 2012 [60] | MDRD eGFR >3.52 | MDRD eGFR <3.52 | mL/min/1.73m^2^ year^-1^ |
| Warner, 2012 [56]^d^ | Rapid GFR^e^ decline^f^ | Stable GFR^e^ decline^f^ | N/A |

^a^P < 0.05; ^b^median ∆eGFR -0.2 mL/min/1.73 m^2^/year; ^c^median ∆eGFR ‑2.6 mL/min/1.73 m^2^/year; ^d^from the CRISP study; ^e^GFR not defined as measured or estimate; ^f^rapid and stable decline not defined.

CKD-EPI, Chronic Kidney Disease Epidemiology Collaboration; GFR, glomerular filtration rate; MDRD, Modification of Diet in Renal Disease; N/A, not applicable.
